# Associations of Maternal Fructosamine before Delivery in Gestational Diabetes Mellitus Pregnancies with Neonatal Glucometabolic Disorders

**DOI:** 10.1155/2022/2478250

**Published:** 2022-11-16

**Authors:** Zhengxia Mao, Ruilin Wu, Huan Yu, Yujiao Zhang, Wenbin Dong, Lile Zou, Xiaoping Lei

**Affiliations:** ^1^Division of Neonatology, Department of Pediatrics, The Affiliated Hospital of Southwest Medical University, Luzhou, Sichuan, China; ^2^Department of Perinatology, The Affiliated Hospital of Southwest Medical University, Luzhou, Sichuan, China; ^3^Department of Histology and Embryology, Southwest Medical University, Luzhou, Sichuan, China

## Abstract

**Background:**

The offspring of pregnant women with gestational diabetes mellitus (GDM) are vulnerable to be glucometabolic disorders. However, to date, few current studies focused on the associations of maternal accumulated glucose exposure before delivery with neonatal glucometabolic disorders and large for gestational age (LGA) infants. This study is aimed at exploring the associations of maternal fructosamine (FMN) before delivery in GDM pregnant women with neonatal glucometabolic disorders in the first 3 days of life and LGA infants.

**Methods:**

The study subjects were the GDM pregnant women, who gave birth in our hospital from September 1, 2018 to January 31, 2021, and their newborns. The maternal FMN adjusted by serum albumin (FMN_ALB_) before delivery was selected as exposure factors. A multivariate logistical regression model was used to calculate the odds ratios (OR) for neonatal glucometabolic disorders, hypoglycemia needing intervention (<2.6 mmol/L), and glucose intolerance (>7.0 mmol/L) in the first 3 days and LGA infants.

**Results:**

In GDM pregnant women, the newborns in the maternal FMN_ALB_ ≥ 75th percentile (≥5.89 mmol/g) group had higher risks in neonatal glucometabolic disorders (aOR 2.50, 95% CI 1.34-4.65, *P* = 0.004) and hypoglycemia (aOR 2.18, 95% CI 1.16-4.10, *P* = 0.016). However, FMN_ALB_ ≥ 75th percentile seemed to be not predictive of the glucose intolerance (aOR 1.76, 95% CI 0.82-3.79, *P* = 0.149) and LGA (aOR 1.56, 95% CI 0.81-3.02, *P* = 0.185). Further, in the sensitivity analysis, the newborns in the maternal FMN_ALB_ ≥ 90th percentile (≥6.40 mmol/g) group also had higher risks in neonatal glucometabolic disorders (aOR 5.70, 95% CI 2.18-14.89, *P* < 0.001) and hypoglycemia (aOR 3.72, 95% CI 1.48-9.31, *P* = 0.005).

**Conclusions:**

The maternal FMN_ALB_ before delivery in GDM pregnant women was a useful biomarker to identify the offspring with high risk of neonatal glucometabolic disorders. However, the association between maternal FMN_ALB_ and the risk of LGA infants was not so strong.

## 1. Introduction

As the most common metabolic complication in pregnant women, gestational diabetes mellitus (GDM) is related to many adverse perinatal outcomes [1]. The newborns of GDM pregnant women are vulnerable to developing hypoglycemia, hyperinsulinemia, and large for gestational age (LGA) infants [1, 2]. Furthermore, it is well known that the fetal insulin resistance is correlated with exposure to high blood glucose in utero, and the newborns of GDM pregnant women have higher fetal insulin resistance [3], so the glucose intolerance and drastic fluctuations of blood glucose can be observed in the newborns of GDM pregnant women in the early postnatal period. For severe or persistent hypoglycemia is related to neonatal brain injury and long-term neurological sequelae [4], most of the previous studies were focused on the prevention and treatment of GDM-related hypoglycemia. However, less attention was paid on the hyperglycemia induced by glucose intolerance in newborns of GDM pregnancies in the early postnatal periods. In fact, severe hyperglycemia was also associated with hypertonic dehydration, intracranial hemorrhage, retinopathy of prematurity (ROP), and other morbidities [5, 6]. In addition, LGA infants and newborn adiposity were associated with obesity, metabolic syndrome, and chronic disease in their later life [7, 8]. Therefore, great importance should be attached to the GDM-related neonatal glucometabolic disorders and LGA infants.

As is well known, the glycemic control in pregnancy is of great importance for GDM pregnant women and their fetuses. Few current studies focused on the associations of maternal accumulated glucose exposure before delivery with neonatal glucometabolic disorders and LGA infants. Hemoglobin A1c, a commonly used biomarker for monitoring glycemic control in type II diabetes mellitus, is inadequate due to anemia or plasma volume expansion during pregnancy [9]. As the nonenzymatic reaction products of glucose with total serum proteins, fructosamine (FMN) can reflect the accumulated blood glucose levels in the past 2-3 weeks. Therefore, FMN has the potential to provide short-term glucose control monitoring where insulin and glucose change rapidly [10] and can be used to evaluate the glycemic control and maternal accumulated glucose exposure in GDM [11]. In the present study, the FMN within 3 days before delivery of GDM pregnant women was used as accumulated blood glucose exposure to explore its relation to neonatal glucometabolic disorders in the first 3 days and LGA infants.

## 2. Materials and Methods

### 2.1. Subjects

In this retrospective study, 264 pregnant women with GDM, who gave birth in our hospital from September 1, 2018 to January 31, 2021, and their newborns were enrolled as the final study subjects.

In the eligible study subjects (*n* = 379), the pregnancies with multiple births (*n* = 24) or combined with hypertensive disorders (*n* = 29), severe cardiac, hepatic, renal, endocrine, and immune system diseases in pregnancy (*n* = 21) were excluded. Besides, newborns with severe structural or chromosomal abnormality were excluded (*n* = 3). Finally, to reflect the accumulated blood glucose levels before delivery better, those pregnancies who missing FMN within 3 days (*n* = 38) before delivery were also excluded. This study protocol was reviewed and approved by the Medical Ethics Committee of our hospital (KY2020252).

### 2.2. Diagnostic Criteria, Exposure, and Outcomes

All data were collected through the hospital information system in this study. According to the criteria of International Association of Diabetes and Pregnancy Study Groups (IADPSG) [12], all pregnant women underwent a 75 g oral glucose tolerance test (OGTT) between the 24^th^ and 28^th^ gestation weeks. GDM was diagnosed based on at least one of the conditions as follows: fasting blood glucose (FBG) ≥ 5.1 mmol/L, 1 h after glucose load ≥ 10.0 mmol/L, or 2 h after glucose load ≥ 8.5 mmol/L [12].

For more than 70% of FMN coming from the productions of blood glucose combined with plasma albumin, the corrected FMN by plasma albumin (FMN_ALB_) can better reflect the correlations between FMN and accumulated blood glucose exposures [13, 14]. Therefore, the maternal FMN_ALB_ within 3 days before delivery was used as the exposure factor reflecting the accumulated blood glucose levels in the past 2-3weeks. For there is no recommended normal value for FMN_ALB_ in GDM pregnant women at present, we used FMN_ALB_ ≥ 75th percentile (5.89 mmol/g) as the cut-off value of poor glycemic control before delivery. Meanwhile, FMN_ALB_ ≥ 90th percentile (6.40 mmol/g) was used as the cut-off value for further sensitivity analysis.

In the clinical practice, all the newborns of GDM pregnant women were fed as early as possible after birth and routinely monitored blood glucose according to the guideline [15]. In this study, the initial neonatal blood glucose was detected by microblood glucose instrument before feeding within 30 minutes after birth using heel blood. For newborns with normal initial neonatal blood glucose, blood glucose was still monitored at the same time in the morning for 3 days.

Neonatal hypoglycemia is defined as neonatal whole blood glucose value < 2.2 mmol/L [16]. However, recent studies showed that neonatal blood glucose < 2.6 mmol/L was associated with short- and long-term morbidities and was widely used as the clinical intervention standard in newborns [17]. In our clinical practice, if the initial blood glucose > 1.4 mmol/L, <2.6 mmol/L, and no clinical symptoms of hypoglycemia, the newborn infants were only fed with milk or breast milk, while other sever cases were immediately intravenously injected glucose 1-2 mL/kg and followed by intravenous infusion of glucose by 6-8 mg/(kg·min) [18]. The blood glucose values were monitored 30 minutes later and once per hour for all infants with abnormal initial blood glucose, until the blood glucose was normal and stable. In the present study, the blood glucose level at any time in first 3 days of life below 2.6 mmol/L was defined as the hypoglycemia needing intervention.

For newborns with normal glucose tolerance, the blood glucose will not exceed 7.0 mmol/L under routine feeding and/or low concentrations of glucose infusion [19]. In this study, under routine feeding and/or glucose infusion below 6-8 mg/(kg. min) in the first three days of life, the blood glucose values exceed 7.0 mmol/L were defined as neonatal glucose intolerance. The neonatal glucometabolic disorders were included both hypoglycemia needing intervention and glucose intolerance.

Based on the fetal-weight reference adapted to Chinese [20], LGA was defined as birth weight by gestational age beyond the 90th percentile.

### 2.3. The Potential Confounding Factors

Referring to previous studies [18, 21–23], the demographic characteristics and potential confounding factors were collected, including maternal age (≥35 and <35 years), III degrees or meconium-stained amniotic fluid, placenta previa or placental abruption, antenatal corticosteroid use, and neonatal gestational age (<32, 32-33, 34-36, and ≥37 weeks), birth weight (<1500 g, 1500-2500 g, and >2500 g), weight for gestational age (small-for-gestational-age, appropriate-for-gestational-age, or LGA), and 1-minute Apgar score (≤7 and >7).

### 2.4. Laboratory Examinations

The FMN and plasma albumin were determined by CL-2000i automatic chemiluminescence immunoassay analyzer (Mindray Bio-Medical Electronics Co., Shenzhen, China). The neonatal peripheral blood glucose values were performed using an i-Sens blood glucose meter and test strips.

### 2.5. Statistical Analysis

SAS 9.4 (SAS Institute, Cary, NC, USA) software was used for data processing and analysis. Multivariate logistical regression model was used to calculate the adjusted odds ratios (ORs) and 95% confidence interval (CI) of maternal FMN_ALB_ before delivery for neonatal glucometabolic disorders, hypoglycemia needing intervention, glucose intolerance, and LGA infants.

## 3. Results

### 3.1. The Baseline Characteristics of GDM Pregnant Women with Different Levels of FMN_ALB_ before Delivery

As shown in Table 1, there were no significant differences in the baseline characteristics between the groups with maternal FMN_ALB_ ≥ 75th percentile and maternal FMN_ALB_ < 75th percentile.

### 3.2. The Associations between the Maternal FMN_ALB_ before Delivery and Neonatal Glucometabolic Disorders

As shown in Table 2, compared to the newborns of the GDM pregnant women with FMN_ALB_ < 75th percentile, the newborns in maternal FMN_ALB_ ≥ 75th percentile group had higher risks in neonatal glucometabolic disorders (aOR 2.50, 95% CI 1.34-4.65, *P* = 0.004) and hypoglycemia needing intervention (aOR 2.18, 95% CI 1.16-4.10, *P* = 0.016). However, no significant increased risk of glucose intolerance (aOR 1.76, 95% CI 0.82-3.79, *P* = 0.149) was observed in infants born to GDM mothers with FMN_ALB_ ≥ 75th percentile.

Further, in the sensitivity analysis, compared to the newborns of GDM pregnant women with FMN_ALB_ < 90th percentile, the newborns in maternal FMN_ALB_ ≥ 90th percentile group also had higher risks in neonatal glucometabolic disorders (aOR 5.70, 95% CI 2.18-14.89, *P* < 0.001) and hypoglycemia (aOR 3.72, 95% CI 1.48-9.31 *P* = 0.005). Similarly, for the risk in glucose intolerance, no significant difference existed between two groups (aOR 2.21; 95% CI 0.80-6.12; *P* = 0.125) (Table 2).

### 3.3. The Association between the Maternal FMN_ALB_ before Delivery and the Risk of LGA

As shown in Table 3, the risk of LGA did not significantly increase (OR = 1.56, 95% CI 0.81-3.02, *P* = 0.185) in infants born to mothers with FMN_ALB_ ≥ 75th percentile.

## 4. Discussion

In the present study, using maternal FMN_ALB_ as an indicator to reflect the short-term accumulated blood glucose levels, the results showed that maternal FMN_ALB_ before delivery was associated with the neonatal glucometabolic disorders in the first 3 days. It suggested that FMN_ALB_ before delivery was an alternative maternal biomarker to identify the high-risk newborns for glucometabolic disorders in GDM pregnant women.

Previous studies have confirmed that the newborns of GDM pregnant women had higher risk of neonatal hypoglycemia, and severe or persistent neonatal hypoglycemia can lead to neonatal brain injury and long-term neurological sequelae [4]. The fetus receives glucose from the mother achieving fetal plasma glucose concentrations 70-80% of the maternal level. As insulin does not cross the placenta, the fetuses of GDM pregnant women have to secrete more insulin to maintain blood glucose in the normal range [24], and the transient high insulin level can cause neonatal hypoglycemia after birth [25]. Our results confirmed that higher maternal FMN_ALB_ before delivery can be a prenatal predictor of hypoglycemia needing intervention in the offspring. In addition, the results from the sensitivity analysis implicated that extremely high maternal FMN_ALB_ should be more vigilant for neonatal hypoglycemia.

Based on the developmental Origins of Health and Disease (DOHaD) theory, exposure to adverse environments, including hyperglycemia, during the early life can lead to fetal metabolic programming [26]. The fetus of GDM pregnant women exposed to intrauterine hyperglycemia and their own hyperinsulinemia can induce abnormal metabolic programming through epigenetic modification such as DNA methylation [27, 28]. And the secretion of insulin from pancreatic *β*-cells and the insulin sensitivity of the peripheral target organs will decrease [29], which can induce the offspring to be insulin resistance, abnormal glucose and lipid metabolism, and overweight/obesity in the short- and long-term after birth [30, 31]. Previous studies have reported that the glucose metabolic disorder of GDM pregnant women was a key factor in the development of insulin resistance in their offspring [32]. Higher maternal blood glucose levels and severe insulin resistance in the third trimester of pregnancy can cause fetal insulin resistance [3, 33]. In this study, neonatal glucose metabolic disorder may be the early manifestation of the abnormal glucometabolic programming in the offspring of GDM pregnant women. Intrauterine hyperglycemia exposure may damage the fetal glucometabolic function, leading to glucose metabolic disorder. Our results showed that, even with no statistical significance for the small sample size, the risk of glucose intolerance was also increased to some extent in the newborns of GDM mothers with higher FMN_ALB_ before delivery. As we all know, in GDM pregnant women, the degree of insulin resistance increases in late pregnancy, and the glucose metabolism also changes rapidly at the same time, so the blood glucose may also changes fast before delivery [34]. As an indicator to reflect the glucose exposure in the past 2-3 weeks, FMN could reflect the changes of blood glucose before delivery. Consistent with our results, we speculate that higher FMN_ALB_ before delivery of GDM pregnant women can reflect the impaired glucose metabolism in the offspring. However, it needs to be verified by a prospective study with larger sample size.

Few studies at present are trying to evaluate the association between FMN before delivery and LGA infants. A recent study revealed that FMN close to delivery was associated with LGA infants in GDM pregnant women [35]. Conversely, our results showed the association of maternal FMN_ALB_ within 3 days before delivery with LGA infants was not significant. There is a rapid growth for fetal during the last trimester of pregnancy when energy stores (particularly glycogen and adipose tissue) are laid down in preparation for birth [36]. However, as an indicator reflecting short-term glucose levels in the past time, the duration for glucose exposure reflected by FMN_ALB_ is too short for fetus to store fat. That may the reason why the association between maternal FMN_ALB_ before delivery was weak in our study.

Our study also has several potential limitations. The selection bias was not avoided from missing FMN_ALB_ before delivery in GDM pregnant women. However, the study subjects were stratified according to the levels of maternal FMN_ALB_ and had no missing data of the neonatal outcomes, which could significantly reduce the selection bias. Secondly, as a tertiary health center in rural area of Southwest of China, for the poor awareness on the self-management of blood glucose, our hospital accepted many GDM pregnancies with poor glycemic control before delivery. Therefore, the incidence of adverse perinatal outcome in our subjects was a bit high; the findings from our study subjects may not be widely suitable for all GDM pregnant women. But in the another hand, it came from the real world of rural area and may give some lights on the management of GDM in the developing country with less medical facility. Moreover, in this study, we only discussed the newborns' glucose metabolism in the early postnatal stage, but we cannot know whether the glucose metabolism is impaired in the future. We guess that neonatal glucose metabolic disorder may be the early manifestation of long-term glucometabolic impairment, which needs more cohort studies to confirm. However, to our knowledge, the present study is the first study on exploring the association between the maternal FMN_ALB_ before delivery and the neonatal glucometabolic disorders in early life.

## 5. Conclusion

In conclusion, our results showed a positive value of maternal FMN_ALB_ before delivery in GDM pregnant women in identifying newborns with high risk of neonatal glucometabolic disorders.

## Figures and Tables

**Table 1:** The baseline demographic characteristics of GDM pregnant women with different levels of FMN_ALB_ before delivery.

	FMN_ALB_ ≥ 75th percentile (*N* = 66)	FMN_ALB_ < 75th percentile (*N* = 198)	*t*/*x*^2^	*P*
Gestational age (*d*)	256 ± 23	255 ± 24	0.51	0.61
Birth weight (*g*)	2921 ± 910	2772 ± 794	1.27	0.21
Maternal age (years)	31 ± 5	32 ± 5	-0.60	0.55
75 g glucose OGTT				
Fasting blood glucose (mmol/L)	5.3 ± 1.0	5.3 ± 1.1	0.03	0.98
1 h after glucose load (mmol/L)	10.3 ± 1.8	10.3 ± 1.9	-0.03	0.98
2 h after glucose load (mmol/L)	8.9 ± 2.0	9.1 ± 2.1	-0.30	0.76
Apgar 1 min ≤ 7, (*n*/*N*, %)	11/66, 16.67%	35/198, 17.68%	0.04	0.85
Apgar 5 min ≤ 7, (*n*/*N*, %)	2/66, 3.03%	7/198, 3.54%	0.04	0.84
Placenta previa or abruption, (*n*/*N*, %)	8/66, 12.12%	30/198, 15.15%	0.37	0.54
III degrees or meconium-stained amniotic fluid, (*n*/*N*, %)	11/66, 16.67%	30/198, 15.15%	0.09	0.77
Antenatal corticosteroid use, (*n*/*N*, %)	14/66, 21.21%	36/198, 18.18%	0.30	0.59
Insulin use during pregnancy, (*n*/*N*, %)	11/66, 16.67%	35/198, 17.68%	0.04	0.85

GDM: gestational diabetes mellitus; FMN_ALB_: the ratio of fructosamine to plasma albumin.

**Table 2:** The associations between the maternal FMN_ALB_ before delivery and neonatal glucometabolic disorders.

	FMN_ALB_ ≥ 75th percentile (*n*/*N*, %)	FMN_ALB_ < 75th percentile (*n*/*N*, %)	OR (95% CI)	*P*	aOR (95% CI)	*P*
Neonatal glucometabolic disorders	37/66, 56.06%	76/198, 38.38%	2.05 (1.17-3.60)	0.013	2.50 (1.34-4.65)	0.004
Neonatal hypoglycemia	29/66, 43.94%	59/198, 29.80%	1.85 (1.04-3.28)	0.036	2.18 (1.16-4.10)	0.016
Neonatal glucose intolerance	13/66, 19.70%	26/198, 13.13%	1.62 (0.78-3.38)	0.196	1.76 (0.82-3.79)	0.149
	FMN_ALB_ ≥ 90th percentile (*n*/*N*, %)	FMN_ALB_ < 90th percentile (*n*/*N*, %)	OR (95% CI)	*P*	aOR (95% CI)	*P*
Neonatal glucometabolic disorders	19/27, 70.37%	94/237, 39.66%	3.61 (1.52-8.59)	0.004	5.70 (2.18-14.89)	<0.001
Neonatal hypoglycemia	14/27, 51.85%	74/237, 31.22%	2.37 (1.06-5.30)	0.035	3.72 (1.48-9.31)	0.005
Neonatal glucose intolerance	7/27, 25.93%	32/237, 13.50%	2.24 (0.88-5.73)	0.092	2.21 (0.80-6.12)	0.125

FMN_ALB_: the ratio of fructosamine to plasma albumin; OR: odds ratios; aOR: adjusted odds ratios; CI: confidence interval. aOR, adjusted for maternal age (≥35 and <35 years), III degrees or meconium-stained amniotic fluid (yes and no), placenta previa or placental abruption (yes and no), antenatal corticosteroid use (yes and no), neonatal gestational age (<32, 32-33, 34-36, and ≥37weeks), birth weight (<1500, 1500-2500, and >2500 g), weight for gestational age (small for gestational age, appropriate for gestational age, and large for gestational age), and 1 minute Apgar score (≤7 and >7).

**Table 3:** the association between the maternal FMN_ALB_ before delivery with the risk of LGA infants.

	FMN_ALB_ ≥ 75th percentile (*n*/*N*, %)	FMN_ALB_ < 75th percentile (*n*/*N*, %)	OR (95% CI)	*P*
LGA	17/66, 25.76%	36/198, 18.18%	1.56 (0.81-3.02)	*P* = 0.185

FMN_ALB_: the ratio of fructosamine to plasma albumin; LGA: large for gestational age; OR: odds ratios.

## Data Availability

The data that support the findings of this study are available from the corresponding author upon reasonable request.
